# GPSNorm: Gaussian Process Spatial Normalization for Spatial Transcriptomics

**DOI:** 10.64898/2026.07.23.740319

**Published:** 2026-07-27

**Authors:** Art Taychameekiatchai, Xiaowei Zhan, Guanghua Xiao, Peifeng Ruan

## Abstract

Spatial transcriptomics technologies enable measurement of gene expression while preserving spatial tissue organization, but they remain highly sensitive to technical variability such as library size differences, slide-level effects, and spatial artifacts. Most existing normalization approaches treat normalization as a preprocessing step and perform downstream analyses on normalized values as fixed inputs, ignoring the uncertainty introduced during normalization. We introduce **GPSNorm** (Gaussian Process Spatial Normalization), a Bayesian spatial normalization framework that jointly models technical variation, spatial structure, and biological signal within a unified hierarchical model. GPSNorm represents gene expression counts using a negative binomial latent Gaussian model whose spatial component is a Gaussian Markov random field approximating a Gaussian process and performs efficient approximate Bayesian inference using the Integrated Nested Laplace Approximation (INLA), producing posterior estimates that propagate normalization uncertainty into downstream differential expression analysis. In simulations anchored to empirical spatial transcriptomics data, GPSNorm accurately recovers spatial technical structure and improves log-fold change estimation compared with existing normalization methods. Applications to three spatial transcriptomics datasets—including human dorsolateral prefrontal cortex Visium data, a GeoMx COVID-19 lung damage study, and the Spatial Organ Atlas kidney dataset—demonstrate improved preservation of biologically expected spatial patterns and marker gene contrasts. These results show that jointly modeling normalization and downstream inference can improve the robustness and interpretability of spatial transcriptomics analyses.An open-source R implementation of GPSNorm is available at https://github.com/Tiny-Quant/GPSNorm .

## Full Text Availability

The license terms selected by the author(s) for this preprint version do not permit archiving in PMC. The full text is available from the preprint server.

